# Epidemic Cholera

**Published:** 1832-01-01

**Authors:** 


### Epidemic Cholera.

Another plague of more gigantic arm
Arose—a monster never known before
Reared from Cocytus its portentous head.
——————— It seemed the general air,
From Pole to Pole, from Atlas to the East,
Was then at enmity with human blood.

The far distant storm, which startled our countrymen on the banks of the Ganges fifteen years ago, and has since ravaged, with devious but too fatal course, every territory from the Straits of Malacca to the Pas de Calais, has at length burst on our shores! In Asia, the fiend was contemplated by us with curiosity—in the wilds of Russia, with suspicion—in Germany, with alarm—but on English soil, with TERROR!

Nothing but lamentable sounds was heard,
Nor aught was seen but ghastly views of death—
*Infectious horror* ran from face to face,
And pale despair.

Whether the pestilence, like the panic, may spread over this fair Island, we cannot tell. We, with many others, hoped and anticipated that the scourge would prove less destructive in England than in other countries; and we do not yet despair that our hopes may be realized. But, as it is not impossible that the enemy may take up his quarters on the banks of the Thames before this paper sees the light—and as the dread, or even the danger, of the epidemic cannot be expected to cease with its annual mitigation or temporary disappearance, we deem it right to select, from the chief records of this disastrous pestilence, such portions of information as may be useful to our readers in the hour of trial. In this article we shall not contend for victory, as too many party-writers do, but for truth and utility. We shall endeavour to steer a middle course between the exclusive contagionists and anti-contagionists—having long been convinced that diseases arising from aerial or terrestrial influences, far beyond our control, have, in the hovels of the indigent, in crowded populations, in concentrated filth, and in the absence of ventilation, taken on a character of infection or communicability which they did not originally possess, and of which they are quickly deprived under opposite and favourable circumstances. The readers of this Journal are well aware that this is the doctrine which we have invariably maintained in respect to fever—and as cholera is probably nothing more than the initiatory stage of some kind of fever, the same doctrine may be safely applied to it. We are free to confess, however, that we are infinitely more favourable to the views of the exclusive anti-contagionists than to those of their opponents, the exclusive contagionists, because we are convinced that their doctrines, on the whole, are infinitely more beneficial to society and to the sick, even if they are wrong, than those of the opposite sect. But we are also convinced that the doctrine of CONTINGENT CONTAGION will be that which is the most safe in practice, and which will be ultimately adopted by the great majority of medical practitioners in these Isles, should they unfortunately have the means of forming their opinions from actual observation.

I.

The first document which we shall notice is that of Professor Lichtenstadt, of St. Petersburg, giving the official Russian Reports of the progress of cholera from Asia to Europe, referring our readers to our Edinburgh contemporary for a fuller account of the Professor's work, if they deem it expedient.

It appears, then, that the first case of cholera occurred on the 26th Aug. 1829, at Orenburg, on the river Ural, about 400 miles north of the Caspian, and 1000 miles north-east of Tabreez, where it prevailed *seven years* previously. On the above day, a man was brought to the military hospital affected with bilious vomiting, diarrhœa, exquisite pain in the abdomen, shrunken features, blue lips, cold extremities, cramps, imperceptible pulse, excessive anxiety. Being treated as for an *inflammatory complaint*, he sunk within twelve hours. In a week after this, a woman died of suspicious symptoms. In another week, viz. on the 8th September, a third case occurred, and terminated fatally in twelve hours.* The case is detailed by Dr. Sokolov, and is as follows:

"The disease began at two in the morning, with a dreadful purging, which returned every minute. Although the weather was cold and wet, the patient went out of doors to obey the calls of nature, barefooted and undressed, and without any precaution. About five o'clock he was without feeling, quite powerless, and affected with constant cramps. At six I found him again sensible, but with sunken pale-blue cheeks, dimness of the eyes, coldness of the feet and hands, and bedewed with clammy sweat. He was tossing about, and complaining of trembling of the hands, a sense of oppression at the pit of the stomach, and intolerable thirst. The vomiting, which, according to his own account, commenced much later than the purging, was at this time less frequent than it had been; but the alvine discharges continued to recur incessantly, and were passed involuntarily. The exhausted, powerless condition of the patient, in particular his completely imperceptible pulse, both at the wrist and over the heart, the stiffness of the limbs, the coldness of the tongue, belly, and præcordia, left me no hope of his recovery. The administration of opium with oil of peppermint and ether checked the vomiting only for a short time; anodyne clysters had no better effect on the diarrhœa; and warm frictions, spirituous drinks, and even the hot bath were resorted to without success to restore the temperature and bring back the pulse. An unsuccessful attempt was in the last place made to draw blood from a vein; and soon afterwards the man expired. Twenty minutes after his last breath, and when the corpse had been already washed and dressed, it was affected all at once with frightful movements. Convulsive motions took place in the hands and feet, like those excited by galvanism, commencing first

---

* None of these people came from infected places, or had communication with any people coming from such. In fact, no trace whatever could be found of the way in which the cholera got into Orenburg. Let the reader remember this when we come to Sir William Crichton's statement.

in a few muscular fibres, especially in the neck and thighs, extending in a vermicular manner, and suddenly producing bending of the head, and agitation and elevation of the feet. These spasms continued with intervals for ten minutes, becoming in the end faint and rare. The same phenomena, though in a less remarkable degree, were observed on another occasion only, but so long as six or seven hours after the termination of the symptoms of the disease."*—P. 115.

Next day two other cases occurred—on the 10th September two more, after which the disease spread rapidly and became prevalent. Between the 9th September, when the cholera commenced, and the 20th November, when it ceased, eleven hundred cases occurred, of which two hundred proved fatal. The population of Orenburg is about eleven thousand souls—so that a tenth part suffered from cholera.

About the 23d of September, or a fortnight after commencing in the capital, it broke out at RASUPNA, 60 *miles West of Orenburg*—and at various dates it shewed itself in various parts of the Orenburgh Government. By the 23d February, 1830, the disease was every where extinct in the said Government.

The symptoms need not be enumerated, as the case already detailed embraces nearly the whole of them—or, at all events, the essential features. The post-mortem appearances are very imperfectly stated by the Orenburg physicians. They agree, however, in saying that marks of inflammation were found in various parts of the intestinal canal. Several of them affirm that these marks were far from being invariably present; and as they do not state the *appearances*, but only the conclusions in their own minds, we may safely doubt whether the said appearances justified the term inflammation. We every day see even eminent medical men, in this country, set down cadaveric changes for living inflammation!

The treatment was grounded on our Indian practice. The Orenburg physicians drew blood from the arm, when it could be done, and prescribed calomel in scruple doses with opium, while every kind of stimulant and caloric was applied to the surface. It is gratifying to find that, where prompt medical assistance was obtained, the mortality of the disease was comparatively small, while in villages and other places deficient in medical skill, the mortality was very great.

We now come to the supposed mode of introduction of the disease, and the manner of its propagation afterwards. We need hardly tell our readers that in India the contagious nature of the disease was denied by 99 out of every hundred medical men in those parts of the world. The Russian Government, however, came to very different conclusions, and early adopted regulations of the non-intercourse kind, together with quarantine, such as are in use against the plague. It was found quite impossible to trace the introduction of the disease into Orenburg. The caravans, the rivers, the roads were all suspected by the contagionists, but none of these could be proved to be guilty. The last caravan arrived thirty-five days before the breaking out of cholera at Orenburg, and the travellers were all in good health. The idea of its being conveyed in the packages of the caravan was

* "These muscular contractions after death were also occasionally observed in the cholera of the East Indies."

too ridiculous to be seriously entertained. Failing in these points, the contagionists *suspected* some neighbouring tribes of half savages, the Kirghis-Kaisaks, dwelling or wandering on the southern and eastern side of the river URAL. These Kaisaks happen to have a very effectual mode of purifying their camps and extinguishing contagious diseases in the bud—namely, by instantly abandoning their sick to the grim tyrant Death, and moving off to a distance of fifty or sixty miles! We may therefore acquit the said Kaisaks of the charge of introducing the cholera into Orenburg.

The foreign introduction of the epidemic being negatived—or at least, "*not proven*," the mode of propagation, after it had once commenced, is the next most important subject of investigation. The majority of the Orenburg physicians who actually witnessed the disease, (and the same will be seen respecting those of Moscow afterwards,) were decidedly against the idea of cholera being communicated from person to person. The general opinion, in Russia, is said to be in favour of contagion. Some attribute it to terrestrial exhalations—a source which our Edinburgh contemporary considers quite preposterous, though it is infinitely the most rational doctrine that has yet been broached—and not the less rational because it was the firm belief of Sydenham himself, no mean observer. Some have thought that a superabundance of the causes of common cholera, with some peculiar state of the constitution, effected the epidemic; while others have attributed it to a certain but incognizable electric state of the air or earth.

It appears that the Orenburg physicians were not only unfavourable, *at the beginning of the epidemic*, to the idea of contagion, but that they encountered facts which were totally irreconcileable with that doctrine. In the *subsequent progress* of the malady, however, they met with facts of a contrary nature—and before the termination of the cholera, many were staggered in their opinions—and a few became decided contagionists. This might be expected; for unbiassed observers, untrammelled by parties and sects, have long ago come to the conclusion that an epidemic, originating in some general causes, may occasionally take on a contagious character in its progress, thus adding to the spread and to the fatality of the disease. But even with this admission, in its fullest latitude, we can see nothing like unequivocal proofs of contagion in the epidemic cholera of Orenburg. Our Edinburgh contemporary, who evinces throughout a strong tendency to the doctrine of contagion, admits that the facts in favour of this doctrine amount only to presumptive, "but by no means to very strong proof." We would say that they hardly amount to presumptive evidence—and therefore we shall not squander away our time or the time of our readers in detailing them. More positive evidence, for or against, will be produced in the sequel. We shall make one short extract from the Edinburgh Reviewer, which will shew the weakness of the contagion cause.

"The last argument in favour of contagion,—the immunity enjoyed by certain small districts in the heart of an infected country, whenever intercourse was cut off with the places where the disease prevailed,—is certainly a fact of much interest and some weight. At the same time so many instances of similar immunity occurred where the fact of free exposure to the supposed contagion could not be called in question; and so many instances also occurred where the disease spread in defiance of the quarantine, that one is tempted to ascribe the escape of the shut up districts, which, after all, were very few in number, to accident, or at least to some other cause than the suspension of human intercourse."* 132.

We have observed that our Caledonian contemporary treats the idea of terrestrial emanations, as the cause of epidemic cholera, with complete contempt. We venture to surmise that the Reviewer has never had opportunities of studying the subject in a practical way—and we are quite certain that he is but little acquainted with medical topography. Thus because Orenburg "is situated in an extensive plain, abounding in undulations to diversify its surface, destitute of lakes and marshes—traversed by clear streams, and not more wooded than is required to beautify the landscape," it is concluded by our sagacious northern critic that the said city is "one of those chosen spots on the globe, which the physician would select for its exemption from all the circumstances of locality that are apt to engender endemic diseases." Yet in the above sentences, he has described, with the most scrupulous exactness, the *Campagna di Roma*—the very focus of pestilence and death! It is really astonishing that a reviewer of the middle of the nineteenth century should entertain the old and trite, and *false* notion that a swamp or a marsh is essential to the production of malaria. Why every medical tyro knows that the most fertile, the most beautiful portions of the earth are the very hot-beds of pestilent emanations. We know too, that localities, which remain for years or even centuries, healthy, suddenly, and without any evident change in appearances, become extremely inimical to human life. How can this possibly be accounted for, except by the supposition that subterranean agents are at work, which cause emanations to rise from the bowels of the earth, without ostensible causes on its surface? These emanations may be wafted along by winds—or they may rise from the earth in a direction contrary to the strongest gales, as they did in India. Such irregular progressions are now taken hold of to support the doctrine of a contagious origin; but they will prove baseless. The cholera is now leaping from city to city, and from shore to shore, in despite of quarantines, and will, we have no doubt, continue to do so.

Another objection urged by the Edinburgh Reviewer to terrestrial exhalation as the primary cause of cholera, is the fact that "elevation above the plain where cholera prevailed did not constitute any protection." He adduces an instance of a village 1400 feet above the plain becoming affected with cholera. Did the learned reviewer never hear of the convent of Camaldoli and a hundred other elevated situations in Italy, where malaria is nearly as fatal as on the plains? Does the reviewer not know that, generally speaking, elevated points in the neighbourhood of malarious plains suffer more than the plains themselves, both in the eastern and western hemisphere? That there was some malarious constitution of the atmosphere prevailing at Orenburg, during, if not previous to the epidemic cholera, is rendered tolerably probable from the following statement of Dr. Onufriev, physician for the circle of Orenburg.

"During the prevalence of the epidemic, there was scarcely a single inhabitant of the City of Orenburg who had not some symptom of disordered digestion. One complained of oppression and pain in the breast; another of head-ache, slight sickness, looseness of the bowels, and the like."

---

* Ed. Journal, July, page 132.

II.

CHOLERA OF 1830. By Sir WILLIAM CRICHTON.

The next document which we shall insert is that of Sir William Crichton, the very title of which shews that Sir William is a party-man, and consequently went in keen search of all facts or opinions that bore on his own side of the question.

"AN ACCOUNT of the Introduction and Progress of the Cholera Morbus in Russia to the end of the year 1830, exhibiting the principal facts which *strengthen the belief of its being a contagious disorder*,* extracted from a Memorial presented to the Medical Council of St. Petersburg by Sir William Crichton, Physician in Ordinary to the Emperor of Russia, &c.

In the Spring of the year 1830 the first authentic accounts of the Cholera Morbus having appeared in Persia were received by the Medical Council of St. Petersburg. It spread itself from the Province of Corasan to Tabrez, the residence of Abbas Mirza, where it made great havoc. A number of the Russian mission to that Prince fell a sacrifice to it, and Prince Dolgorouky, the Russian Minister at the same Court, was saved with great difficulty from a serious attack of it.

In the beginning of July the disease penetrated the Russian Provinces of Schirvan and Bacon, from whence it spread by land as far as Tifflis, and by sea from the port of Bacon to Astracan.

It broke out in these two last-mentioned towns nearly at the same time; that is to say, on the 20th of July. It appears from the accounts we have received, that neither at Tifflis nor at Astracan any precautions were taken to prevent its spreading further, probably from its not having been thought contagious, so that it extended with rapidity from Tifflis throughout Georgia and the Province of Caucasus, always following the principal roads.

At its first appearance at Astracan (which took place soon after the arrival of a vessel from Bacon, on board of which eight men had been seized during the voyage, with the Cholera, and had died of it,) thousands of people, employed in navigating the Volga, together with fishermen of that river, made their escape from the town, the first re-ascending the Volga, the others going up the river Pural or Jaik. The disorder showed itself at Gowrieff on the 26th of July, at Ouraesk on the 3d of August. Let us now observe its course along the Volga, the great line of communication by which the disease penetrated into the interior of Russia. At Senolayerisk the Cholera broke out the 22d or 23d of July, at Krasmoyar the 25th of the same month, at Tzaritzen the 6th of August, at Donbooka and Saratoff the 7th, at Khoalinsk the 19th, at Lamara and Neigni Novogorod the 27th, at Kostroma the 3d of September, at Zaroslaff the 6th, and at Rybinsk the 11th of the same month. In all those places the first victims of the disease were either navigators of the Volga, or individuals arrived from places where it already raged.

A Cossack, who had been sent the beginning of August from the station named Katchalinskara on the Don, to buy provisions at Donbooka on the Volga, died of the Cholera on the 7th, after his return to the station. After that circumstance, the malady spread successively through the different Cossack villages along the river Don; without enumerating all these, suffice it to say, that the first deaths from Cholera at Novetcherkash, the principal town of the Cossacks, took place

* What business had any man searching after truth to prejudge the whole inquiry in the very title of the paper? But this is the way with all partizans. When they set out on their travels they take good care to keep one eye shut!

on the 18th of August, at Rastaff, its ravages began some days later, and that on the 9th of September it had penetrated as far as Jaganrog. A great number of persons of all ranks escaped from Saratoff, (a town containing 40,000 inhabitants,) and took refuge in the next government of Peusa, but the Cholera did not fail to follow them, and commenced its depredations in that province on the 13th of August.

The first death at Kasan was on the 9th of September, an individual who had come there from Nigni Novogorod.

It has not been exactly ascertained who was the first individual who died at Moscow of the disorder, as most of the Physicians of that city did not believe in its having visited them, consequently no exact information was then taken of those who were attacked with it. There is, however, reason to believe that the first victim was a student, who had leave of absence from Saratoff, and whose servant died on the road thence to Moscow.

### *Of the Causes.*

Although there is still a difference of opinion among physicians on this subject, the Medical Council of St. Petersburg is obliged to acknowledge that the exciting cause of this disorder (and the only one well proved,) is a specific contagion, less virulent, perhaps, than that of the plague, and requiring a certain pre-disposition in the human body for its development, but which contagion certainly exists; numerous proofs of this fact were presented by the epidemic of 1829 and 1830.

1st.—The progress of the Cholera along the high roads.

2d.—The remarkable circumstance that *the first who died of it wherever it appeared, were individuals who arrived from some infected place.*

3d.—That the places where immediate precautions were taken, were not attacked by it, as for example, the small town of Sarepta (inhabited by a colony of Moravians), situated on the high road from Astracan to Tzaritzen, and only twenty-five versts from the latter place. Also, several farms and country-houses near Astracan, and German colonies in the government of Saratoff, the military school of cadets at Moscow, &c. around all of which the malady raged furiously.

4th.—That in Moscow, which contains at least 200,000 inhabitants, there have been since the 16th September, up to the present instant, 6th January, only 6,000 or 7,000 sick, or one twenty-ninth of the population; an incredibly small number, if we look for the general cause of Cholera Morbus in atmospheric influence.

### *Means of Prevention.*

Considering the contagious nature of the disease, and the rapidity with which it spreads, the Government, at the recommendation of the Medical Council, ordered quarantines to be formed on the frontiers of every province in which the disease raged; and afterwards entirely to surround all places where it existed. After the experience of the epidemic in 1829, the Medical Council found that a quarantine from fourteen to twenty-one days was sufficient to ascertain the state of health of any person coming from an infected place, instead of confining him six weeks, as in the case of the plague. *During the whole course of the years 1829 and 1830, there is not a single instance that can be relied on of the contagion being communicated by articles of dress or furniture, &c.**

Fumigations made with chlorines, were generally employed as means of dis-

---

* If the disease were really contagious, would not clothes be very apt to convey it from place to place. Would the clothes of a typhous or a variolous person be so safe and innocent?

infection; but experience does not justify us in speaking positively as to their efficacy."

On this document of Sir W. Crichton we shall make few comments, for, in truth, we consider it not only of no value, but *worse* than useless. When we find in it so many gross errors as to facts, within investigation, we may fairly conclude that in various other parts, at the truth or error of which we cannot get, there is no lack of special pleading—to give it no worse name. Thus let the reader peruse the second reasons which Sir William triumphantly adduces as proof that the cholera morbus everywhere originated in contagion. "The first who died of it *wherever it appeared,* were individuals who arrived from *some infected place.*" Now the first victims to the disease at Orenburg came from no infected place, and no trace of contagious introduction could ever be discovered. The second reason is equally futile, because it assumes for fact what is *not fact.* In a *fortnight* after the cholera broke out at Orenburg it started forth all at once at Rasupna, 60 miles from Orenburg!! Truly this choleric traveller must have worked hard to travel 60 miles in a fortnight, infecting all the towns and villages on the "great roads." The infection along the road, however, existed only in Sir William's own brain, where there is, no doubt, an ample manufactory for all such kinds of stuff. Sir W. tells us that it has not been ascertained who was the first individual who died of cholera at Moscow; but he soon makes up for this want of information, by *supposing* that the first victim was a student, who had leave of absence from Saratoff! Alas! alas! for medical science, when such bolsterings up of preconceived hypotheses are sent forth into the world as historical facts.

But Sir William's third reason in favour of contagion is the best of all. Only one in 29 of the inhabitants of Moscow suffered from the disease! This reason is too ridiculous to deserve notice. Does Sir William remember that almost the whole of the inhabitants of Orenburg and Moscow laboured under indisposition of some kind, at the time of the cholera? Was this not a pretty certain sign of some general cause in the air? Upon the whole, Sir William's document is a very poor specimen of the arguments adduced in favour of the contagious *origin* of cholera morbus.

### III.

We shall next record the report of Dr. Albers, a Prussian physician, at the head of a commission sent by the Prussian Government to Moscow, to ascertain the nature of the cholera morbus. This document is dated the 21st of March, 1831.

"On the nature of the distemper, and the question which is so very important to us, in how far the Cholera is contagious, there prevails as yet the greatest diversity of opinions. Under the supposition which we look upon as erroneous, that this question is to be decided on the facts hitherto known, which are connected with the nature of recorded infections, two parties have formed themselves, those of the contagionists and anti-contagionists, the former particularly among the authorities and physicians of St. Petersburg, *and the latter among the faculty and inhabitants of Moscow, who almost all of them strenuously maintain that Cholera is not contagious.* Both parties cite facts which are met with point blank contradictions by the opposite party, whence the unprejudiced inquirer finds it as yet impossible to form a conclusive judgment. The vastness of the

Empire, the very unsatisfactory manner of the few reports sent in, the uncertainty of depositions, influenced frequently by personal motives, and the almost totally interrupted correspondence by letters, offer so many obstacles to inquiry, that even with the best intention it is often only possible in part to overcome them.

When the Cholera first reached Moscow, *all the Physicians of this city were persuaded of its contagious nature, but the experience gained in the course of the epidemic, has produced an entirely opposite conviction.* They found that it was impossible for any length of time completely to isolate such a city as Moscow, containing 300,000 inhabitants, and having a circumference of nearly seven miles (versts ?), and perceived daily the frequent frustrations of the measures adopted. During the epidemic it is certain that about 40,000 inhabitants quitted Moscow, of whom a large number never performed quarantine ; and notwithstanding this fact, *no case is on record of the Cholera having been transferred from Moscow to other places,* and it is equally certain that in *no situation* appointed for quarantine, *any case of Cholera has occurred.* That the distemper is not contagious, has been yet more ascertained by the experience gathered in this city. In many houses it happened that one individual attacked by Cholera was attended indiscriminately by all the relatives, and yet did the disease not spread to any of the inmates. It was finally found that not only the nurses continued free of the distemper, but also that they promiscuously attended the sick chamber, and visited their friends without in the least communicating the disease. There are even cases fully authenticated that nurses to quiet timid females labouring under Cholera, have shared their beds during the nights, and that they, notwithstanding, have escaped uninjured in the same manner as physicians in hospitals have, without any bad consequences, made use of the warm water used a moment before by Cholera patients for bathing.

These and numerous other examples, which during the epidemic, (we ought perhaps to call it endemic,) became known to every inhabitant of Moscow, have confirmed the conviction of the non-infectious nature of the disease, a conviction in which their personal safety was so much interested.

It is also highly worthy of observation, that all those who stand up for contagion, *have not witnessed* the Cholera, which is therefore especially objected to their opinion by their opponents. But in the very difference of the conviction of those who have to combat the violence of the distemper, and are likely to be more impressed by the facts, and of the conviction of such persons as can observe only at a distance, and are therefore more unbiassed judges of the results, will perhaps be found materials for the solution of a question so much controverted. The same was the case on occasion of the question relative to the Yellow Fever. It was only after a calm examination of all the results, that it became possible to refute the error of those physicians who had collected their experience during their daily and fearless intercourse with the distemper, and had arrived at the conviction of its non-contagious nature.

In the instance of the Cholera, the question becomes more difficult of decision; because if the Cholera be at all contagious, of which I myself am not doubtful, in spite of all that is maintained here, such contagion differs from the nature of all known contagions, and seems to approach nearest to that of the *Typhus.* With whatever obstinacy the correctness of the facts is disputed by the anti-contagionists, it still appears highly probable, that the Cholera may be communicated by persons proceeding from one place to another, and may lay the foundation of a fresh epidemic, if circumstances favour the communication.

It is greatly to be lamented, that neither of the contending parties is able to produce such authentic documents, and to set on foot such investigations on the spot, as would silence every contradiction ; for as the state of the question now is, we must be satisfied with probabilities.

Only one point seems to be completely made out by by testimonies innumerable; namely, *that the Cholera is not communicated by articles of merchandize, or by any inanimate objects.* This principle, as I have already had the honour of reporting sometime ago, has been adopted by the public authorities of St. Petersburg, and been acted upon now for nearly three months, without any sinister consequence having ensued. The only quarantine establishment still kept up is between Moscow and St. Petersburg; every traveller, after staying there for a fortnight, may proceed without further detention; all mercantile commodities and effects pass without being stopped.

On our journey hither, we met many thousands of sledges loaded with goods, going from Moscow to St. Petersburg. As the rates paid for carriage are extremely reasonable, any stoppage in their conveyance would prejudice the merchant; hence the carriers, as I myself saw, proceed no further than the barriers of the quarantine establishment, and remain there, as far as their persons are concerned, and their sledges alone pass through, which, being met on the other side by their partners or servants, are taken on without hindrance. The result of my own daily experience, therefore, perfectly agrees with the above stated principle; namely, notwithstanding all my inquiries, *I have met with no instance which could render it at all probable, that the Cholera is disseminated by inanimate objects.*"*

In the above document it will be seen that all the *facts* which Dr. Albers brings forward, are against contagion; while, nevertheless, he doubts not the contagious character of the malady, thus laying himself open to the opprobrium which he himself has recorded, namely, "that all those who stand up for contagion, *have not witnessed the cholera.*"

But how does Dr. Albers make it out that cholera assimilates nearest to *typhus,* when he tells us that articles of cloathing and other inanimate objects are incapable of harbouring or transmitting the contagion? Is he not aware that the fomites of typhus are very readily harboured in cloathing? It is, indeed, abundantly evident that Dr. Albers stated his *facts* faithfully; but fashioned his *creed* according to the instructions of his masters! God grant that both *facts and opinions* may not be moulded according to certain pre-ordained forms, even in this country!

### IV.

We entreat the attention of our readers to the following document, entitled—

"REPORT ON THE CHOLERA MORBUS, DISCUSSED AND AGREED TO IN THE EXTRAORDINARY COMMITTEE ESTABLISHED AT MOSCOW BY ORDER OF HIS MAJESTY THE EMPEROR.

An Extraordinary Committee, composed of the most eminent public officers, has been established at Moscow, by order of His Imperial Majesty, for the purpose of discussing the expediency of a general purification of all merchandize in Moscow after the cessation of the Cholera Morbus in that capital. The Committee in consequence proposed the following question to the Members of the Provisional Medical Council:—Can goods or merchandize communicate the Cholera Morbus? and in case of an answer in the affirmative, What is the degree of the intensity of the contagious principle? The result of the examination of the opinions of the twenty-four Members of the Council is, that three of them admit,

* Parliamentary Papers.

it is true, the possibility of contagion by means of goods and merchandize, but under certain conditions ; eighteen entirely reject it. One member admits it : but, from the experiments which he has made, he does not think fumigation necessary. Another member recommends the adoption of this measure, but only for the purpose of tranquillizing men's minds. Finally, another declares that he knows no fact which proves the communication of the Cholera Morbus through the medium of material objects. He thinks, however, that it will be useful to apply fumigation to some kinds of merchandize, such as cloth, by employing chlorate of lime, and merely to expose all other goods to the air. The Committee having given to the examination of this subject all the attention which the importance of the question demanded, and which the orders with which they were honoured by his Imperial Majesty enjoined them, have unanimously come to the following conclusions :—

1st. The quarantine regulations relative to the purification of goods and merchandize have been established from observations made on the Plague; they have therefore been adopted, under the present circumstances, entirely by conjecture. Nevertheless, it was impossible to avoid adopting these regulations as long as the contagious influence of the Cholera Morbus, and the means by which it spreads itself, were not yet determined by accurate observations. It is necessary, then, to replace these ancient regulations by others more appropriate to the new disease, and equally founded upon evident facts.

2d. It has not hitherto been possible to collect in any place in the empire so many accurate observations on the Cholera Morbus, nor to unite on one spot so many able physicians as at Moscow, where, during the three last months, more than 7,500 sick were treated by the care of the Provisional Medical Council, and 52 bodies dissected. It is only then in this capital that the examination of all the opinions pronounced on the Cholera Morbus, opinions hitherto conjectural, contradictory, and founded on a small number of equivocal or ill observed facts, can be proceeded in with the best chance of success.

3d.—Although the members of the Provisional Medical Council have not pronounced an unanimous opinion relative to the communication of the Cholera Morbus by means of goods and merchandize, nevertheless *the majority at least, pronounced against this hypothesis, and the opinions of the minority destroy themselves.* They offer many contradictions, and do not correspond with known facts. For example, a Member advances 'that the virus of the disease *(virus morbifique)** 'of the Cholera Morbus is not so subtle as that of the Plague ;' he then adds, 'that is proved by a great number of examples, that persons in health have been 'attacked by the epidemy, from having made use of beds or clothes which had 'belonged to victims of the Cholera.' In fine he maintains, 'that it is more 'by analogy than from positive experiments, that it may be affirmed that goods 'which communicate the Plague would equally communicate the Cholera Morbus.' If this member merely founded his opinion on the analogy which he believes to exist between the Plague and the Cholera, it would follow, that he ought not to have mentioned the number of examples which he might have observed, even on the supposition that a physician who only treated 300 cases of Cholera, may have been able to collect an infinite number of observations. In fine his assertion on the analogy between the Cholera and the Plague, is in contradiction with the difference which he himself says ought to exist between the contagious principles of these two diseases. The second member who declares the Cholera Morbus contagious, expresses himself in these terms : 'This epide-

* This is improperly translated. A "morbific virus," and the "virus of a disease," are quite different things. Thus, a morbific virus may be malaria ; but the virus of a disease, is an emanation from the body of a patient labouring under the *effects* of malaria.—Ed.

'mic disease cannot arise either from a change of temperature or from the na-
'ture of the food, or from confined habitations, or from bad clothing;' while he subsequently refutes himself, by saying, 'aged people or those who lead an irre-
'gular life, those who are subject to catch cold, or to stomach complaints, or in
'fine, who are not regular in their diet, are more exposed than others to the ac-
'tion of the Cholera Morbus.' The third member of the minority gives the following example in support of his conviction of the possibility of the communication of the Cholera Morbus by goods and merchandize; 'An individual who
'was suffering of a quinsy was attacked by cramp in his legs, from having bathed
'his feet in a vessel which had been used to empty the bath of a *Cholériste*.' Still if in truth the Cholera Morbus spreads itself in this manner, it is not probable that such a case should be observed but once during the treatment of more than 7,500 sick. It must then be concluded that the cramp was brought on by some other cause which has escaped the investigation of the physician.

4th.—On the contrary the opinion of those who do not admit the possibility of contagion, by means of material objects, has for its support both the majority of voices and the scrupulous observance of facts. The members of the Medical Council have been convinced by their own experience, as also by the reports of the physicians of the hospitals, that, after being in frequent and even habitual communication with the sick, their own clothes have never communicated the disease to any one, even without employing means of purification. Convalescents have continued to wear clothes which they wore during the disease, even furs, without having them purified, and they have never had a relapse. *At the opening of bodies of persons who had died of the Cholera, to the minute inspection of which four or five hours a day for nearly a month were devoted, neither those who attended at these operations, nor any of the assisting physicians, nor any of the attendants, caught the infection, although, with the exception of the first day, scarcely any precautions were used.* But what appears still more conclusive, a physician who had received several wounds in separating the flesh, continued his operations, having only touched the injured parts with caustic. A drunken invalid having also wounded himself, had an abscess, which doubtless showed the pernicious action of the dead flesh, but the Cholera Morbus did not attack him. In fine, foreign Savans, such as Moreau de Jonnes and Gravier, who have recognized, in various relations the contagious nature of the Cholera Morbus, do not admit its propagation by means of goods and merchandize.

5th.—A member of the Committee justly observes that the trade of Moscow, after having languished at the time the Cholera Morbus reigned there with all its force, recovered its activity in November, when the epidemy was becoming weak, and that since the first cold, there has been a considerable circulation of merchandize, as well of that manufactured at Moscow as imported into it. Moreover, if the germs of the contagion had been concealed, their action would have shown itself either in individual cases, or in the return of the ravages of the epidemy through the town, and in the increase of the number of victims. This not having taken place, it is conclusive that the disease does not spread itself through the medium of material objects.

6th.—On the contrary supposition, the result would be, that since 1,500 of the 7,500 above-mentioned sick were taken care of at home, and in consequence exempt from the active superintendence of the medical police, the articles (effects) with which their houses were furnished, and with which the sick were in constant contact, would rather tend to spread the contagion than merchandize deposited in magazines which had not been touched by any one. It would become then much more necessary to purify effects shut up in every house in Moscow, than the merchandize. The almost total cessation of the epidemy evidently shows that no general contagion has taken place by means of the above-mentioned effects, the purification of which would be besides very difficult and even impracticable. Even after the Plague, all the houses at Moscow were not purified, but only those in which sick were known to have been, or of which the inhabitants were dead.

7th.—Even supposing that, which however is only conjecture, the Cholera Morbus was effectually propagated by merchandize brought from the fair of Nizni Novgorod, it would result as this merchandize has been spread not only in Moscow but as far as St. Petersburg and a number of other towns, and in great part distributed to the consumer, either that the contagion did not exist in the merchandize at Moscow more than at St. Petersburg, or that it is necessary to purify St. Petersburg, and the other towns which have received the merchandize from the fair of Nizni Novgorod, in the same manner as the city of Moscow would be purified.

8th.—But even if, without attending either to the evidence of the proofs which establish the impossibility of contagion by merchandize, or to the want of accurate observations, which might serve to establish the contrary, it should be decided through excess of precaution to purify all the merchandize in Moscow, this measure would not be the less followed by consequences which demand all the attention of Government. The alteration of the colours and of the lustre of the merchandize would produce a sensible diminution in their value, and the loss of considerable capitals; trade would for a long time stand still, many establishments of industry would be ruined, and thousands whose livelihood depends upon the manufactories would be reduced to a frightful state of misery.

9th.—From these considerations the Committee have concluded, in conformity with the order of His Majesty the Emperor, of the 25th of August last, that it is not necessary to subject the merchandize to fumigation in those places where the Cholera Morbus has existed." 14.

The above document will speak for itself, and we apprehend that it will form a tolerable set-off against the contagionists who have no experience of the epidemic themselves.

V.

We shall now proceed to take notice of two communications to Government from Dr. Walker. The first letter, dated Moscow, 15th March, needs little notice, as it is chiefly occupied with marches and counter-marches in pursuit of cholera—with but indifferent success. At length, however, the Doctor came up with the expiring enemy at Moscow, and had an opportunity of seeing a few cases of the disease there—but he was very doubtful whether they were *all* (14 in number) really cases of cholera. Dr. Walker affirms that the symptoms of the disease, and the appearances on dissection, "are exactly the same as those described in the official reports from the medical boards of the three Presidencies of India, &c." But as Dr. Walker did not see the cholera of India, he cannot be considered as a perfect judge. How can a man be certain of the identity of two diseases, when he has only seen one of them? We positively assert, and we shall illustrate this in the sequel, that the graphic description of Indian cholera is inadequate to convey an exact idea of the disease. The eye must be added to the ear, to enable a man to form a clear conception of the malady. Be this as it may, Dr. Walker seems rather puzzled in making up his opinion on the subject of contagion—and explains himself so badly, that he is obliged to add a postscript to enable the reader to comprehend his creed. The following portion of Dr. W.'s second letter, dated St. Petersburg, 29th April, 1831, contains all the information which it is necessary to lay before our readers.

"I hasten to communicate the results of my own observations, and of the information which I have been able to collect from others respecting that most important point, the contagious or non-contagious nature of the disease.

In Moscow by far the greater part of the medical men are of opinion that the disease is not contagious, but produced by some peculiar state of the atmosphere, not cognizable by either our senses or by instruments; *that this was proved by almost every person in the city feeling during the time some incovenience or other,* which wanted only the exciting cause of catching cold, or of some irregularity in diet, to bring on Cholera; that *very few* of those immediately about the patients were taken ill. That persons *had put on the clothes of patients who were very ill or had died of Cholera, had lain in their beds, or even alongside of corpses, had bathed in the same water where very bad Cholera patients had been bathed just before, and that none of these persons were taken ill.* That a strict investigation had been made into what were reckoned *the First Four cases occurring in Moscow, and that it was proved that they had neither themselves been in any infected place, nor had communication with any one coming from such a place.** I confess, however, I am not quite satisfied with this, because,—1st. In a place of such an extent as Moscow, every one can conceive it perfectly possible that there *may have been cases before these without its being known,* and I *have the* CERTAINTY *myself of its being possible,* for it occurred to me while I was there, to know of Four cases that had never been reported to the police, nor seen by a medical man.†

2d. One of the Four cases had only a few days arrived from Simbirsk, so that he might have brought the *seeds* of the disease with him, because the disease was in the *district* of Simbirsk in the end of August, and he died in Moscow, I think on the 16th of September.

3d. This investigation was not made by medical men, but through the police, which I do not reckon the proper method, as the people are so afraid of them, that it is next to impossible to get at the truth."

"With respect to the possibility of the disease being communicated by clothes or goods, no cases have as yet come to my knowledge sufficient to prove it. I have heard of several instances brought forward in support of the opinion, but they are not fair ones; as in all of them the persons had either come from places where the disease was, or it was already prevalent in the place where they were living. And by far the most general opinion, even among the contagionists, is, that it is only through the medium of the body that it is propagated.

So that the result of what information I have been able as yet to collect on the subject, is, that I believe it is capable of being conveyed from one place to another by men, *although it cannot be considered completely proven;* while, although there is not evidence sufficient to prove its communication by clothes or goods, still we cannot say that it is impossible. *See* Postcript."

"Although it does not admit of legal proof, yet there is no doubt that, from the great difficulty, or indeed impossibility, of keeping quarantine strictly in such a great extent of country as that where the disease prevailed last summer and autumn, numbers of persons and quantities of goods from infected places, evaded the quarantines, and came even to this city, but fortunately without bringing the disease."

"Although such is my opinion, yet I should not conceive it necessary to have any Quarantine for vessels arriving in England from Russia, unless the disease

---

* Compare this with the positive assertion of Sir William Crichton to the contrary!

† Thus we see the contagionists always substituting suppositions for facts. The first four cases that were visible and tangible, were free from suspicion of contamination; but then Dr. Walker *supposes* that other cases *might have happened* prior to the four, and thus the whole business *might have* resolved itself into contagion, instead of atmospheric influence!!!

prevailed at the place of loading or in the neighbourhood, and even then perhaps only in the event of any person on board having had the disease during the passage. For although there are not as yet any observations regarding the length of time that the disease may lie dormant in the system, the general opinion is, that it does not probably exceed fourteen days, and therefore there would be little or no risk in admitting a vessel that had been at least fourteen days on her passage, without having any sick on board. Persons however might get in a considerably shorter time from here to England, by the steam-boat to Lubeck, if there should not be any quarantine there."

"*Postcript.*—I find the expressions I have made use of here, have appeared to others not to convey quite the meaning I myself attach to them. I intended to say that I myself am *convinced* of the contagious nature of the disease, but that the *proofs* of its transmission from one individual to another are not *quite perfect* as yet. And believing so, I cannot of course be without some apprehension that it may also be conveyed by clothes and other articles, which have been in more immediate contact with the sick, although the proofs of this are, as yet, still more defective. It is a disease *sui generis*, and must have its own laws as well as the plague, typhus fever, and other contagious or infectious disorders, but these laws we do not yet sufficiently know. Its attacks seem to be favoured by depressing passions, especially fear of the disease, great fatigue, low bad living, bad air in crowded dirty dwellings, drunkenness. I have been informed that in Austrian Gallicia, where it had made its appearance, a better diet furnished to the lower orders at the expense of the government, seems to have contributed as much as any other measure to prevent the spreading of the disease.

Thos. Walker."

### VI.

We are now arrived at the point where defensive measures were taken up by our own Government; and Sir William Pym naturally leads the van on this important occasion.

"Letter from Sir *W. Pym* to the Clerk of the Council, suggesting immediate measures against the introduction of the Cholera.

Sir, London, 7th June, 1831.

In consequence of the intelligence received this day, relative to the spread of Cholera in Russia and Poland, but more particularly on account of its having reached the port of Riga, I have thought it my duty to state to you, for the information of the Lords of His Majesty's Council, my opinion as to the necessity of immediately having recourse to more decided measures against the introduction of the disease into the United Kingdom. With this view I beg, 1st, to propose, that the quarantine be imposed upon all vessels coming from the Baltic, the Hanseatic towns, and the ports of Mecklenburg: 2d, That pilots and officers of Customs be strictly ordered by the proper authorities to carry all vessels liable to quarantine, having clean bills of health, to the regularly appointed quarantine stations: 3d. That all vessels having foul bills of health, or arriving without bills of health, from ports where Cholera prevails, be ordered by pilots and officers of Customs to proceed to Cromarty Bay, to Stangate Creek, or to Milford Haven: 4th, That experienced persons be appointed to superintend the vessels under quarantine at Grimsby, at the quarantine anchorage in the Firth of Forth, and in the Firth of Tay, near Dundee, and at Cromarty Bay, if this last should be thought necessary; and that medical attendants should be appointed at those stations for the purpose of inspecting the crews and passengers, and reporting upon their state of health previously to their being admitted to pratique: 5th, *That an order be issued, prohibiting altogether the importation of every description of woollen rags; and as it was considered that certain articles, hemp, flax, wool, &c. &c. generally termed susceptible, (with respect to their liability to retain the*

*contagion of plague, and of other contagious and infectious diseases,) might, in consequence of the arrangements made respecting them by order of the Russian Government, for the purpose of dividing them into different qualities, be imported from the ports of Russia with perfect safety, so long as the population at those ports continued free from disease, it now becomes a question, and a very important one to be decided, whether goods so deemed susceptible can be imported with safety (without quarantine purifications) from ports where Cholera prevails;* taking into consideration the importance of the subject, and the very great responsibility attached to me individually, in my official situation, I beg to submit, whether it would not be most desirable, as well as a great satisfaction to the Public, that the Lords of the Council should call upon Sir Henry Halford and the College of Physicians for their opinion and report upon this most important point.

Should their opinion be in favour of the purification of goods of any kind, it would be desirable, in addition to the regulations before proposed, that all Vessels from the Baltic, having goods on board requiring purification, should be ordered to proceed either to Inverkeithing Bay in the Firth of Forth, to Stangate Creek, Milford Haven, Liverpool, or Holy Loch in the Clyde, those being the only ports where there is any Lazaret accommodation. I have the honour to be, &c.

(signed) *W. Pym,*

Superintend. Gen. of Quarantine." 15.

The above letter of Sir William Pym, together with all the other documents which we have noticed, being submitted to the president and fellows of the College of Physicians, the following answer was returned, in which it will be seen that the College demurred to the 5th proposal of Sir William Pym, respecting the prohibition of rags, and the purification of other articles of merchandize.

" OPINION of Sir *H. Halford*, Bart. and other Physicians, on the Nature and Symptoms of the Cholera.

Having no other means of judging of the nature and symptoms of the Cholera than those furnished by the documents submitted to us, we, the undersigned Physicians, have come to the conclusion, after most careful consideration, that the disease is of an infectious nature, and therefore does require that all persons coming from an infected quarter should be placed under a Quarantine of at least Fourteen days.

With respect to merchandize, as we do not find any proof in these papers that the disease has been propagated by means of inanimate objects,* and as the Russian Government was induced by the strong representations of the Extraordinary Committee appointed ' to determine upon the propriety of a general purification of all the merchandize in Moscow, after the extinction of the Cholera in that city,' to give up the plan of fumigating such articles of commerce, we cannot bring ourselves *to believe it necessary to adopt the 5th Article of Sir William Pym's recommendations, but in the propriety of his Four first proposals we entirely acquiesce.*

(signed) HENRY HALFORD, {President of the Royal College of Physicians.
THOMAS TURNER,
WILLIAM MACMICHAEL,
June 9th, 1831. FRANCIS HAWKINS.

*P. S.*—The date of the Fourteen days, we presume, is to be reckoned from the time of leaving the infected place, and on condition of no person being sick of this malady during the voyage.

(signed) HENRY HALFORD." 16.

* " See Dr. Alber's Report."

Now we perfectly concur in the demurrer of the College respecting Sir William Pym's 5th proposition, because they acted on the evidence of facts submitted to their inspection; but how are we to account for the sudden change in the councils of the College, when, a few days afterwards, we find the demurrer abandoned, and a recommendation to prohibit and fumigate merchandize, according to Sir William's fifth proposition?

"College of Physicians, June 15th, 1831.

TO THE LORDS OF HIS MAJESTY'S MOST HONOURABLE PRIVY COUNCIL.

WE, the President and Fellows of the Royal College of Physicians in London, having carefully considered all the Papers which have been transmitted to us by order of Your Lordships, have agreed upon the following Report:

That the Evidence submitted to us, in the Documents sent to the College by the Lords of His Majesty's most Honourable Privy Council, warrants an opinion that the disease called Cholera in Russia is communicable from one person to another.

Although these documents contain some statements which lead us to doubt whether this infection is conveyed by merchandize or not, yet until we have further information, *we recommend that articles of merchandize admitted into this Country, from infected places, should be submitted to the usual regulations of Quarantine.*

In the name of the College,

(signed) *Henry Halford*, President.

(signed) *Francis Hawkins*, Registrar." 17.

We puzzled our brains, in vain, to account for this revolution of opinion in the course of six short days, during which no new information was received. Did the great captain of quarantine throw his yellow standard into the scale, in imitation of a famous captain of antiquity, who threw his sword there, and thus make it kick the balance in favour of contagion?

The short report of the College above inserted being unsatisfactory to the Government, a more detailed statement of the *reasons* for the conclusions of the College was demanded: accordingly a long letter was forwarded to Government, embodying chiefly the account given by Sir William Crichton, on which we have already commented.

It is not a little remarkable that, in all these accounts, there is no allusion made to the epidemic cholera of 1829, which ravaged the city and government of Orenburg. How can we depend on Sir William Crichton's information, when he tells us that it was in the Spring of 1830 that the first accounts of the cholera morbus reached the medical council of St. Petersburg!! Professor Liechtenstadt has, as our readers will perceive, published a detailed account of "the cholera, as it appeared in Russia in the years 1829 and 30." These striking defects, and the unequivocal bias, or rather inveterate prejudice, with which Sir William writes, take away all confidence in his *opinions*.

### VII.

Since the above was written—now many months—we have listened to the reports, and perused the statements made through various channels, respecting the cholera morbus—all of which have only tended to strengthen our conviction that the disease is non-contagious, except under circumstances of *filth and foul air;* and that all our quarantine regulations (more especially internal quarantine) are worse than useless. We see that almost the whole of the Continent has thrown off this INCUBUS, convinced of its inefficacy as to prevention, and of its detriment, by stagnating commerce and keeping the minds of men in perpetual dread of contagion. How did cholera originate in Hamburgh? Was it carried thither by caravans, diligences, or steamers? No, indeed! It broke out "in a miserable resort called the DEEP CELLAR, frequented by beggars, vagrants, and other abandoned subjects, of both sexes, and to this profligate class of people it has hitherto been chiefly confined." We find that, in ten days after the breaking out of this dreaded scourge, the people of Hamburgh talked and cared ten times less about it than before its advent, and while they were hemmed in by cordons and quarantines! Waiting, then, with equanimity the approach of cholera from the banks of the Wear to the shores of the Thames, we shall dedicate a portion of this article to select notices of some of the many works on that disease which have issued from the press, even since the publication of our last number, and with the weight of which our table actually groans. Many, indeed most, of these publications will require but little notice here, being re-publications of old materials, got up for the gratification of that public excitement which naturally prevails when a terrible calamity impends over our heads. In a disease, too, where the cause, the nature and the treatment are quite undecided, there is ample scope for hot-headed individuals to rush into the field, each pregnant with the important conclusions to which he has come—very often without any other *data* than the conceptions of his own brain!

